# Supplementary material for: Genome Editing to Generate Sake Yeast Strains with Eight Mutations That Confer Excellent Brewing Characteristics
Source: Cells. 2021 May 24;10(6):1299. doi: 10.3390/cells10061299 (PMC8225151; doi:10.3390/cells10061299)
Supplement: Supplementary file 1 [file cells-10-01299-s001.zip › Supplementary Figures 0512.pdf]

Supplementary Figures

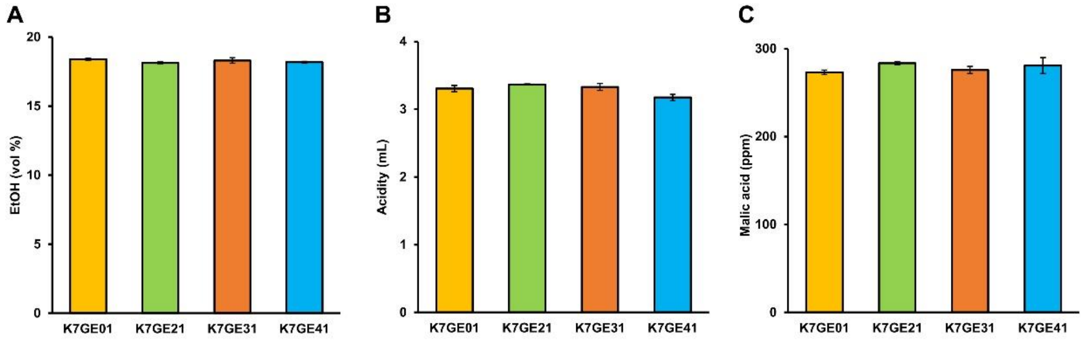

Figure S1. Measurement of ethanol (A), acidity (B), and malic acid (C) in sake made with genome-edited strains. Error bars indicate standard error (n = 3). Acidity value indicates volume of 0.1N NaOH required for neutralization of sake samples.

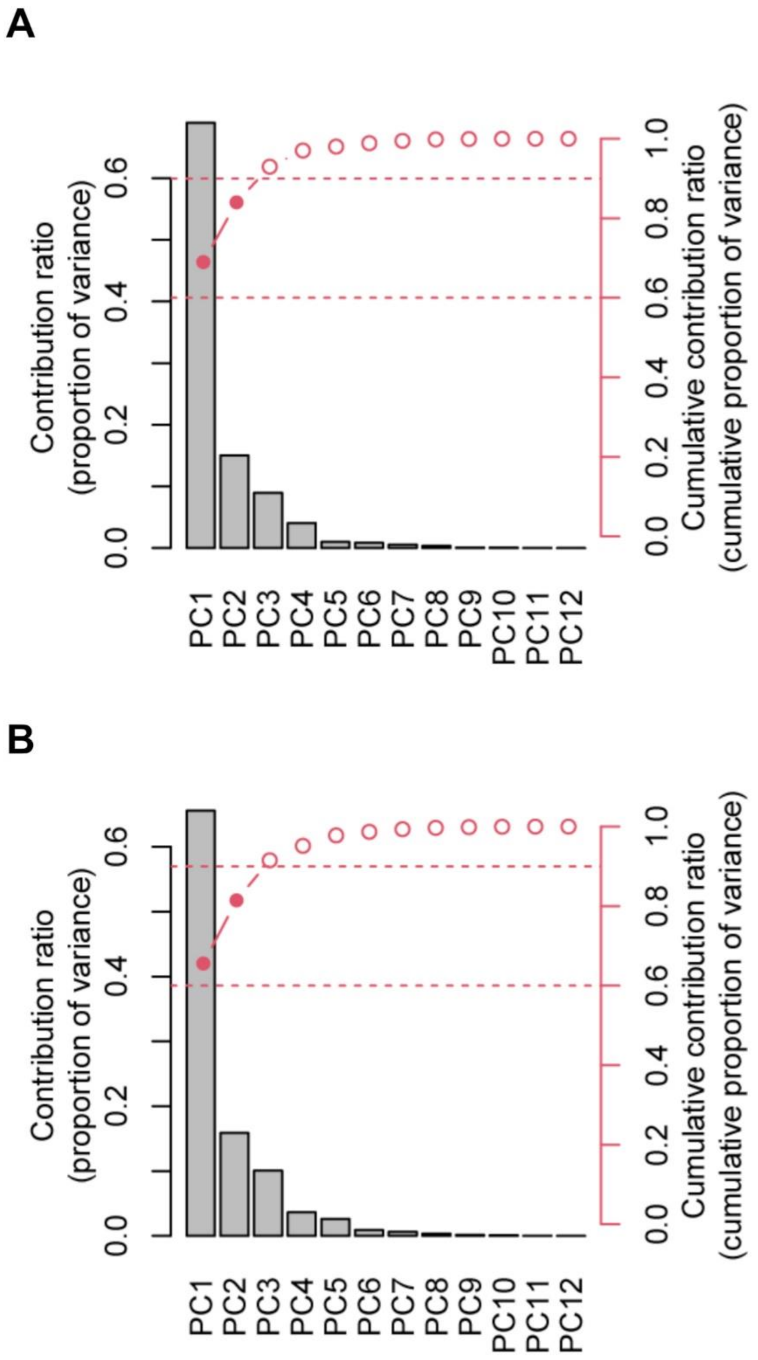

Figure S2. Cumulative contributions of principal components after PCA with 19 (A) and 16 (B) components.

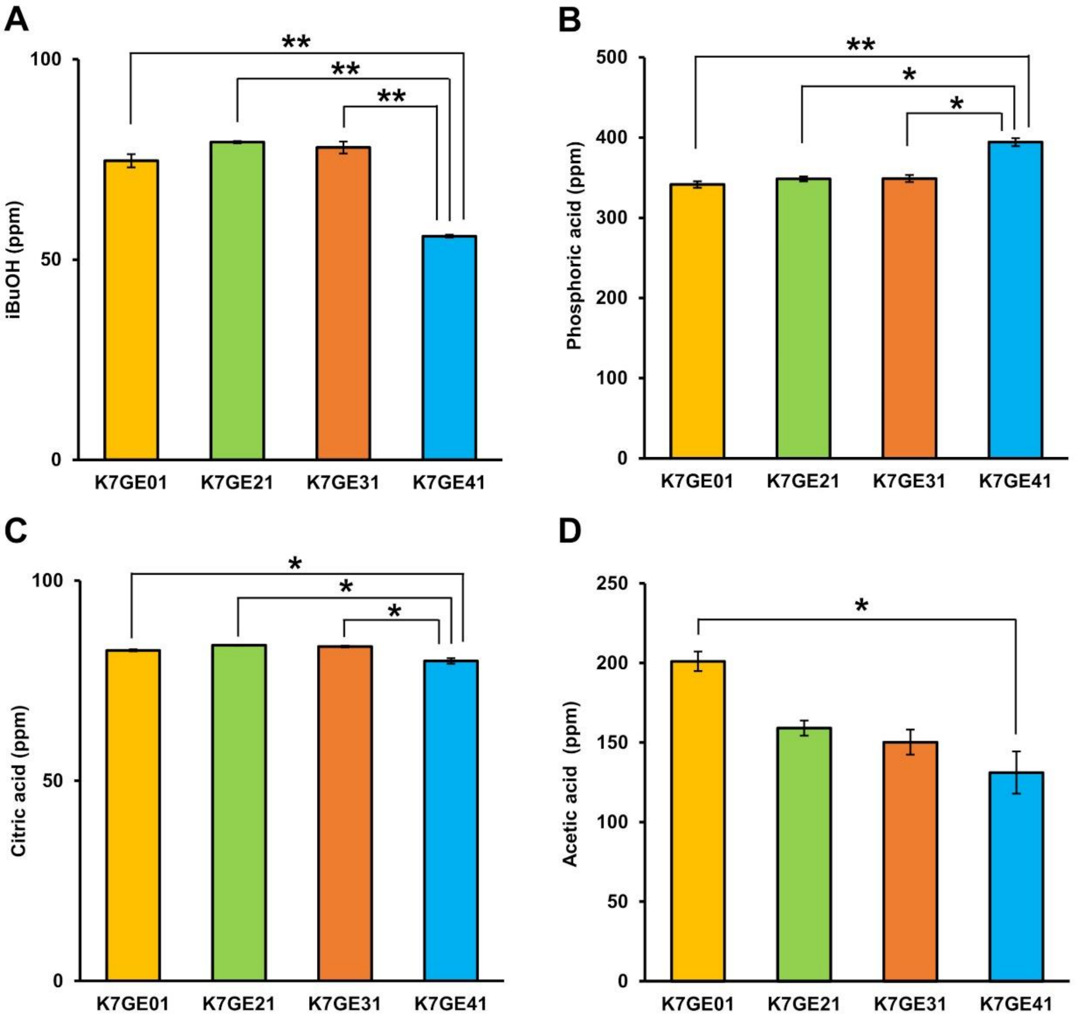

Figure S3. Measurement of isobutyl alcohol (A), phosphoric acid (B), citric acid (C), and acetic acid (D) in sake made with genome-edited strains. Error bars indicate standard error (n = 3). Asterisks indicate Tukey's multiple comparison test (* $p < 0.05$, ** $p < 0.01$).

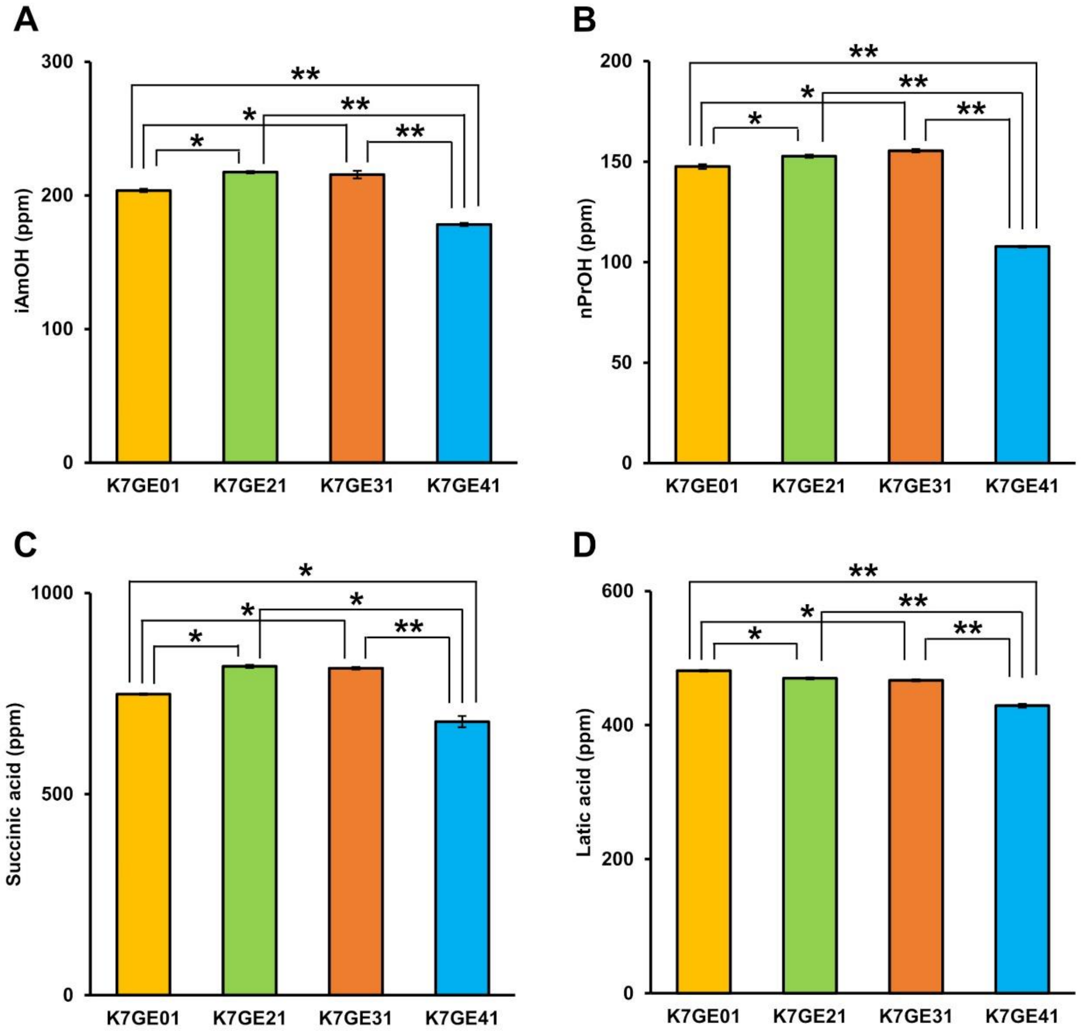

Figure S4. Measurement of isoamyl alcohol (A), 1-propanol (B), succinic acid (C), and lactic acid (D) in sake made with genome-edited strains. Error bars indicate standard error (n = 3). Asterisks indicate Tukey's multiple comparison test (* $p < 0.05$, ** $p < 0.01$).

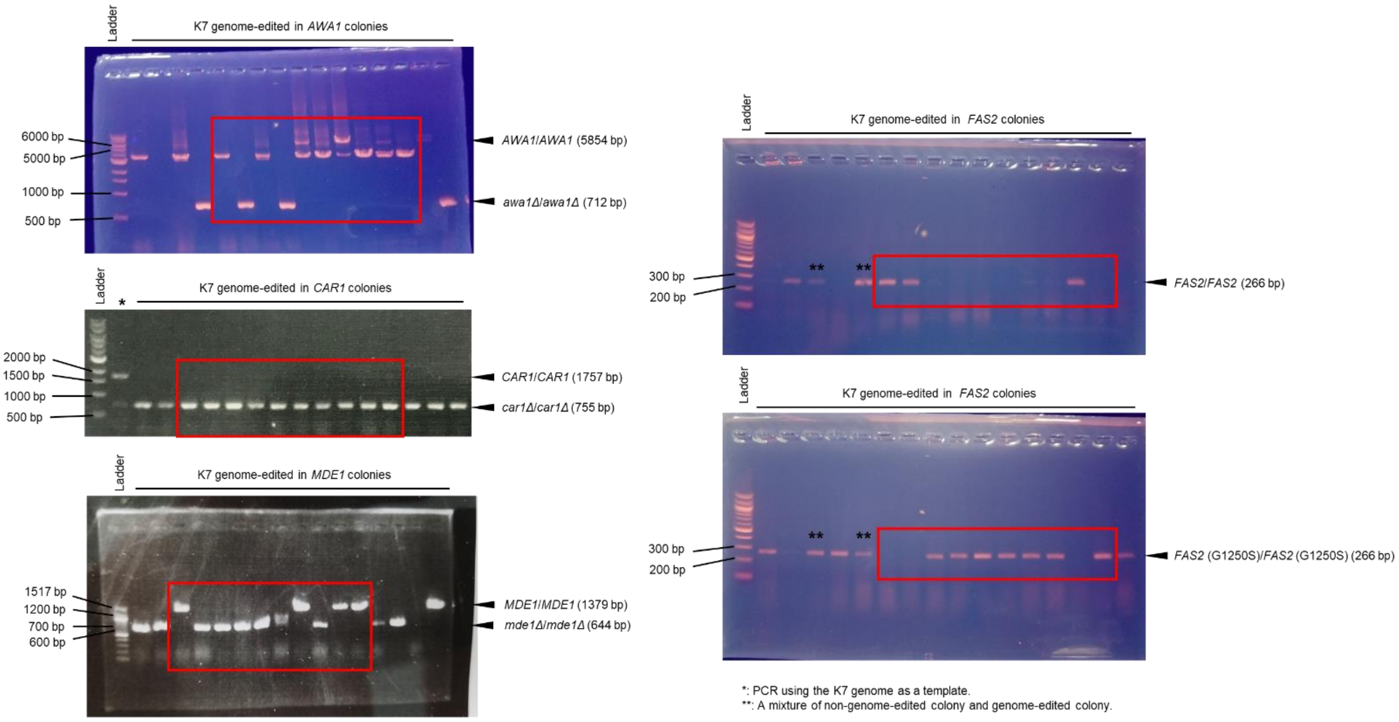

Figure S5. Raw data of Figure 2B.
